# Parasites as environmental bioindicators and climate-sensitive sentinels in arid ecosystems: a one health synthesis with a focus on Saudi Arabia

**DOI:** 10.3389/fvets.2026.1978111

**Published:** 2026-10-20

**Authors:** Hanadi B. Baghdadi, Mohamed Abdelsalam, Mohamed Abdo Rizk

**Affiliations:** 1 Department of Biology, College of Science, Imam Abdulrahman Bin Faisal University (IAU), Dammam, Saudi Arabia; 2 Department of Aquatic Animal Medicine and Management, Faculty of Veterinary Medicine, Cairo University, Giza, Egypt; 3 Department of Clinical Sciences, College of Veterinary Medicine, King Faisal University, Al-Ahsa, Saudi Arabia

**Keywords:** arid ecosystems, bioindicators, climate change, environmental parasitology, host–parasite dynamics, marine parasites, one health, parasite ecology

## Abstract

Parasites integrate environmental conditions across trophic levels and may therefore provide sensitive, mechanistically interpretable signals of ecosystem change. This narrative synthesis evaluates the potential of parasites and parasite communities as environmental bioindicators and climate-sensitive sentinels, with particular emphasis on arid ecosystems and Saudi Arabia. We integrate evidence from terrestrial, aquatic, marine, and zoonotic systems to examine how warming, drought, hydrological change, salinity, pollution, land-use transformation, and host-mediated vector movement influence parasite occurrence, transmission, phenology, community composition, and contaminant accumulation. The evidence supports two complementary bioindicator functions: accumulation indicators, in which parasites concentrate environmental contaminants, and effect indicators, in which parasite prevalence, phenology, richness, or community structure responds to environmental disturbance. Saudi marine systems provide particularly strong examples, including trace-metal accumulation by acanthocephalans and nematodes, community-level changes associated with habitat degradation, and seasonal transmission of Kudoa sp. in *Priacanthus tayenus*. Terrestrial and waterborne systems provide additional candidate signals through tick phenology and the distribution of Cryptosporidium and Giardia. However, the current evidence base is dominated by cross-sectional studies, heterogeneous diagnostic methods, and geographically fragmented datasets; consequently, parasites should be regarded as promising candidate sentinels rather than validated early-warning indicators. We identify priority research needs and propose a One Health surveillance architecture integrating standardised parasitological sampling, environmental and meteorological measurements, molecular identification, remote sensing, livestock-movement data, and multivariate community analysis. Long-term, coupled datasets will be essential to distinguish climate-driven signals from concurrent anthropogenic pressures and to translate parasite bioindication from an ecological concept into an operational monitoring tool for arid and peri-arid regions.

## 1 Introduction

Parasites constitute the most species-rich trophic strategy on Earth, with at least 50% of all living species being parasitic in some form (59), engaging virtually every free-living organism in networks of exploitation, co-evolution, and ecological regulation through approximately 75% of all food web links (60). Despite their ubiquity, parasites remain among the most underappreciated components of biodiversity assessments and environmental monitoring programmes (1, 62). This neglect is paradoxical: parasites integrate signals from multiple trophic levels through their complex life cycles that depend on various vertebrate and invertebrate hosts (63), respond sensitively to thermal, hydrological, and chemical perturbation as demonstrated through meta-analyses of pollutant effects (64), and transmit across species boundaries in ways that simultaneously reflect ecosystem integrity (61) and carry direct public health implications through their role in infectious disease dynamics (2, 65). The concept of parasites as bioindicators—organisms whose presence, abundance, or community composition reveals information about environmental conditions—has gained traction in aquatic toxicology and marine ecology (3, 4), yet its rigorous application to climate change, particularly in arid terrestrial and coastal ecosystems, remains fragmented and incompletely validated.

Arid and semi-arid regions are disproportionately sensitive to climatic shifts. In the Middle East and North Africa (MENA), projected temperature increases of 2–4 degrees Celsius by mid-century, declining rainfall, expanding desertification, and intensifying dust-storm frequency place pressure on ecosystems already operating near physiological thresholds (5, 6). Saudi Arabia exemplifies these pressures: a hyper-arid country covering approximately 2.15 million km2, it hosts residual schistosomiasis transmission foci, endemic cutaneous leishmaniasis, persistent malaria ecology in its southwestern highlands, and a rapidly urbanising population that concentrates zoonotic interfaces between humans, livestock, and peridomestic wildlife (7). Projections under high-emissions scenarios (RCP 8.5) indicate that up to ~30% of parasitic worm species globally may face extinction risk by 2070 when host-driven co-extinction dynamics are incorporated—an upper-bound estimate contingent on the concurrent realisation of both host and parasite range losses; habitat loss alone is projected to account for 5–10% extinction risk (8). Remaining species are projected to be redistributed into novel host-environment configurations. At the same time, Saudi Arabia’s Red Sea and Arabian Gulf coastlines support marine ecosystems under simultaneous thermal, salinity, and anthropogenic stress, where fish parasite assemblages exhibit proposed bioindicator value that warrants systematic validation (9, 10).

This review synthesises evidence across terrestrial, aquatic, and zoonotic domains to evaluate how parasites function as candidate early bioindicators of climate and environmental change, with Saudi Arabia and the broader Arabian Peninsula as the regional focal case. We adopt a One Health perspective, recognising that parasite signals in wildlife, livestock, and aquatic hosts carry direct implications for human health. Our objectives are: (i) to consolidate the mechanistic understanding of how arid-zone environmental drivers shape host–parasite dynamics; (ii) to evaluate the proposed bioindicator value of specific parasite–host systems in Saudi Arabian terrestrial and marine environments; and (iii) to identify critical research gaps and propose a surveillance framework for parasite-based environmental monitoring in arid regions (Figure 1).

**Figure 1:**
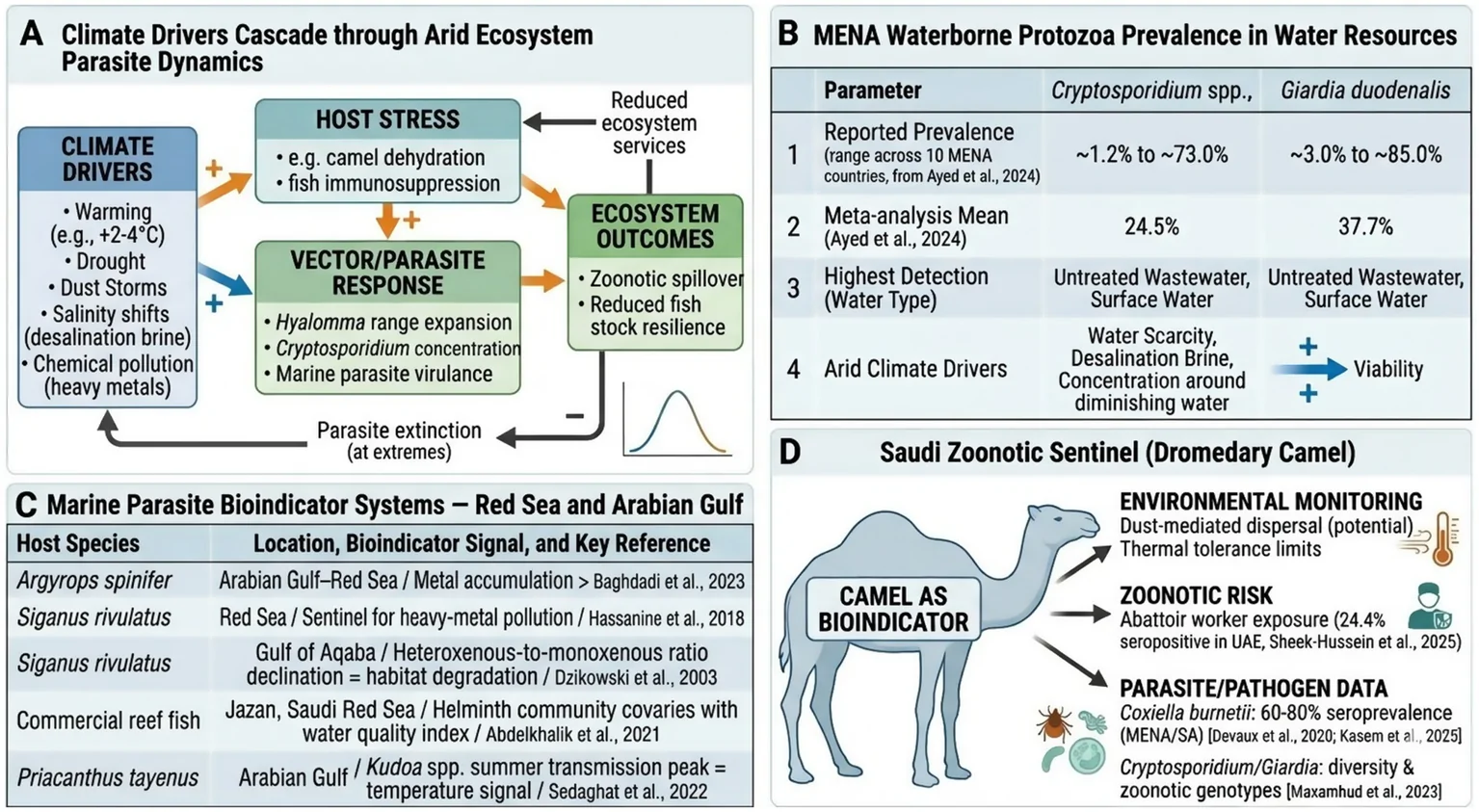
Conceptual synthesis of climate-driven parasite dynamics in arid and coastal ecosystems, with empirical data from Saudi Arabia and the MENA region. **(A)** Climate Drivers Cascade through Arid Ecosystem Parasite Dynamics. Key climate stressors (warming +2–4 degrees C, drought, dust storms affecting host immunological status, desalination-derived salinity shifts, and chemical pollution from trace metals) act positively on host stress and vector/parasite responses through several directionally context-dependent pathways, amplifying zoonotic spillover risk and reducing fish stock resilience across a range of host–parasite thermal optima. At thermal extremes, the same drivers impose negative pressure, resulting in localised extirpation of thermally sensitive taxa, consistent with unimodal thermal performance curves described by Gehman et al. (29) and Murdock et al. (28). **(B)** MENA Waterborne Protozoa Prevalence in Water Resources. Meta-analysis data from Ben Ayed et al. (51) across 10 MENA countries showing pooled detection rates of approximately 24.5% for Cryptosporidium spp. and approximately 37.7% for Giardia duodenalis in surveyed water resources. Highest detection occurs in untreated wastewater and surface water. Water scarcity and ephemeral hydrological foci may concentrate oocysts and cysts at remaining water points, with persistence dependent on extended cyst/oocyst osmotic resilience rather than confirmed halotolerance enhancement under brine conditions (a testable hypothesis requiring empirical validation). **(C)** Marine Parasite Bioindicator Systems — Red Sea and Arabian Gulf. Summary of empirically demonstrated fish host–parasite bioindicator systems: trace metal bioaccumulation in *Sclerocollum saudii* (Pb up to 289x host muscle) (12); trace metal accumulation via ingestion in Hysterothylacium spp. nematode larvae in *Psettodes erumei* from the Persian Gulf (14); helminth species richness and diversity indices demonstrating statistically significant correlation with physicochemical water quality parameters at Jazan coast (47); longitudinally confirmed directional trophic shifts in heteroxenous/monoxenous ratios (10); cross-sectional patterns consistent with such shifts at Gulf of Aqaba reef sites (9) (requiring multivariate longitudinal validation); and the first record of Kudoa sp. temperature-dependent phenological peak (7.6% summer; 0% winter) in *Priacanthus tayenus* from Jubail, Saudi Arabian Gulf (48). Gefen et al. (15) caution that pooled h/m indices require site-level stratification and multivariate frameworks (nMDS/PCoA) for valid interpretation. **(D)** Saudi Zoonotic Sentinel: Dromedary Camel as a Candidate Sentinel Host. The dromedary camel integrates environmental monitoring signals, zoonotic risk, and parasite/pathogen data. Individual-level *C. burnetii* seroprevalence: 50.4% in Makkah Province camels (53) (ELISA); herd-level prevalence: 92.9% nationally (54). UAE abattoir: 52.9% in slaughtered camels; 24.4% in abattoir workers (56). Cryptosporidium/Giardia: diverse zoonotic genotypes confirmed in Algerian Sahara dromedaries (36).

### 1.1 Review approach

The article is a transparent narrative evidence synthesis. Detailed information on literature identification, search concepts, screening, evidence selection, and qualitative appraisal is provided in the new “Literature Search and Evidence Synthesis” subsection above. Because no formal systematic-search protocol or quantitative meta-analysis was undertaken, findings are interpreted qualitatively and reported estimates are not pooled by the present review.

## 2 Parasites as bioindicators: conceptual foundations and functional categories

### 2.1 Literature search and evidence synthesis

The review was informed by structured searches of PubMed/MEDLINE, Web of Science Core Collection, Scopus, and Google Scholar, supplemented by backward and forward citation tracking of key reviews and primary studies. The search timeframe extended from database inception through September 2026, with the final update performed during the present revision. Search concepts combined terms related to parasites and parasite communities (parasite*, helminth*, protozo*, tick*, vector*, host–parasite), environmental change (climate change, warming, drought, salinity, pollution, water quality, land-use change, desalination), bioindication (bioindicator*, sentinel*, environmental monitoring), and the regional context (Saudi Arabia, Arabian Peninsula, Gulf, Red Sea, MENA). Studies were retained when they provided primary or authoritative evidence relevant to parasite occurrence, abundance, community structure, transmission, phenology, contaminant accumulation, or parasite-mediated environmental signals in relation to climate or other environmental drivers, bioindicator/sentinel function, or One Health implications. Priority was given to peer-reviewed primary studies and authoritative reviews providing direct evidence from Saudi Arabia or the Arabian Peninsula; comparative studies from MENA and other arid, marine, or climate-sensitive systems were retained when they provided mechanistic or methodological context. Studies were not excluded solely because they originated outside Saudi Arabia, but their geographic origin and evidentiary role are explicitly identified in the revised synthesis. Studies not addressing the relationship between parasites (or parasite communities) and environmental conditions, bioindication, or the One Health context were not retained for the substantive synthesis. No language restriction was used as a formal eligibility criterion; however, the practical evidence base was predominantly English-language literature. Screening was performed iteratively by title and abstract relevance, followed by full-text assessment for relevance to parasite ecology, environmental drivers, bioindicator function, or One Health implications. Duplicate or clearly non-relevant records were not retained for synthesis. Because this is a narrative synthesis rather than a systematic review, no formal risk-of-bias instrument or quantitative study-quality score was applied. Instead, evidence was critically appraised qualitatively according to study design, geographic relevance, directness of evidence, temporal resolution, diagnostic approach, and whether the reported association supported mechanistic inference. Quantitative prevalence estimates are therefore reported descriptively and are not treated as pooled estimates generated by the present review.

Useful bioindicator organisms generally satisfy several criteria: ecological sensitivity to the stressor of interest, measurability through standardised methods, broad geographic distribution enabling spatial comparison, and interpretability within a causal framework linking environmental change to biological response (1, 2). Parasites satisfy these criteria with particular strength because their life cycles depend on the co-occurrence of definitive hosts, intermediate hosts, and suitable abiotic conditions for free-living transmission stages. When any link in this chain is disrupted by temperature shift, habitat loss, or hydrological alteration, parasite prevalence, intensity, community composition, or transmission phenology changes in detectable ways (4, 11). A critical but often neglected step in applying parasite community data as bioindicators is the functional categorisation of the bioindicator signal. Sures (3) and Sures et al. (2) distinguished between accumulation indicators—parasites that passively absorb and store trace metals or organic pollutants at concentrations exceeding ambient levels, without necessarily suffering immediate physiological toxicity—and effect indicators—parasites whose community structure, population size, or species richness changes measurably in response to the stressor. Both categories are represented in Saudi Red Sea systems, but their physiological mechanisms and monitoring implications differ fundamentally.

In the Red Sea, acanthocephalans such as *Sclerocollum saudii* function as accumulation indicators through tegumental absorption: lacking a digestive tract, they absorb ambient toxins directly through their integument, achieving lead bioconcentration factors up to 289-fold above host muscle tissue (12). By contrast, raphidascaridid nematodes such as Hysterothylacium spp. accumulate trace metals primarily through the ingestion route, sequestering metals from prey tissues via their digestive system; consequently, metal loads in Hysterothylacium spp. reflect dietary rather than purely ambient environmental exposure (13, 14). Lumping these taxa into a monolithic bioindicator class obscures the mechanistic pathway of metal accumulation and reduces the interpretive precision of monitoring data. Community-level effect indicators, exemplified by shifts in the heteroxenous-to-monoxenous parasite ratio, operate through an entirely different pathway—the collapse of trophic network complexity—and require multivariate rather than univariate analytical frameworks (10, 15).

Lafferty (16) was among the first to formalise the bioindicator logic, arguing that parasites provide sensitive indicators of human impacts on the environment precisely because their complex life cycles amplify subtle ecological signals across trophic levels. Marcogliese (1) subsequently demonstrated that parasite communities in aquatic systems simultaneously reflect food-web structure, biodiversity, and environmental stress, making them integrative rather than single-stressor indicators. Lafferty (17) linked parasite diversity to host biodiversity at the community scale, demonstrating that biodiversity loss causes predictable declines in parasite diversity—a pattern with feedback implications in which the erosion of specialist parasites may paradoxically amplify generalist parasite transmission in residual hosts, directly relevant to simplified host communities in degraded arid-zone habitats. Palm et al. (18) confirmed in tropical mariculture that parasite diversity indices track long-term environmental modification, while Lewis et al. (19) and Diamant et al. (20) established that disaggregation of parasite communities by life-history type—particularly the separation of macroparasites from microparasites—substantially improves the sensitivity of pollution monitoring.

In terrestrial systems, tick phenology tracks warming with high fidelity: documented northward range expansions of *Ixodes ricinus* in Europe and altitudinal shifts of Hyalomma species in the Mediterranean broadly parallel isothermal displacement (21, 42). Vector-borne parasites can therefore act as climate sentinels whose distributional changes presage broader ecological reorganisation (11). Critically, parasites may signal environmental change earlier than their hosts: free-living hosts may tolerate moderate stress through behavioural thermoregulation or physiological acclimatisation, whereas parasites with narrow thermal tolerance windows or obligate dependence on specific intermediate hosts often respond with threshold-like shifts in transmission success (22). This asymmetry positions parasite community data as a potentially leading indicator of ecosystem trajectory—a property of value for early-warning surveillance, though one that requires longitudinal validation before operational deployment in Saudi arid settings.

## 3 Climate change and host–parasite dynamics: mechanistic frameworks

The mechanistic pathways through which climate change alters host–parasite interactions operate at multiple biological scales and are directionally context-dependent and highly variable, not uniformly pro-parasitic (6, 23). Marcogliese (24) established that changes in climate or local environment are bound to affect levels of parasitism through shifts in host phenology, intermediate host availability, and free-living stage survival. Subsequent syntheses extend this framework to show that aquatic metazoan parasites respond to temperature alongside indirect effects mediated through dissolved oxygen, ocean acidity, salinity, host immune competence, and the abundance and distribution of suitable intermediate hosts (22, 66). Atroch et al. (25) comprehensively reviewed climate-change impacts on fish and shellfish parasites, finding that rising temperatures can accelerate virulence and reproduction of direct-life-cycle parasites while simultaneously compromising piscine host immunocompetence via immunometabolic trade-offs; however, warming may disrupt complex-life-cycle parasites whose intermediate and definitive hosts exhibit discordant thermal ranges, illustrating why directional predictions require system-specific rather than universal assumptions.

Tomamichel et al. (26) conducted a meta-analysis of 266 effect sizes from 52 empirical studies on harvested aquatic species, finding that higher temperatures generally increased parasite-induced host mortality; critically, the magnitude of this relationship varied substantially by host order and parasite type—Salmoniformes experienced greater temperature-amplified mortality than the cross-study average, while bacterial parasites showed reduced infected-host mortality with warming. Studer et al. (27) experimentally demonstrated the threshold character of thermal effects in a controlled intertidal trematode-amphipod system: within the 20–25 degrees Celsius range, cercarial output and infectivity peaked (maximum parasite-induced risk), whereas at temperatures exceeding 30 degrees Celsius, transmission was reduced despite direct host thermal mortality increasing. This unimodal structure—a thermal optimum beyond which transmission declines—is a consistent feature of parasite ecology (28) and precludes simplistic extrapolation from mean temperature increases to transmission outcomes.

Gehman et al. (29) experimentally demonstrated that host and parasite thermal performance curves interact jointly: warming of approximately 2 degrees Celsius can drive local extirpation of thermally sensitive parasite taxa in some coastal systems, while other host–parasite pairs experience amplified transmission under the same increase. Dahlin et al. (30) extended this framework through modified Ross-MacDonald models to show that host availability and biting tolerance interact with temperature to shift the thermal optimum for mosquito-borne parasite transmission by up to 5 degrees Celsius. This 5-degree shift represents a fundamental epidemiological reconfiguration of the transmission curve: in arid zones where host biodiversity simplification accompanies habitat degradation, the effective thermal optimum for vector-borne parasite transmission may therefore differ substantially from the thermal optimum measured in species-rich reference systems, providing a mechanistic basis for why vector-borne diseases may emerge at unexpected temperatures in ecologically simplified Arabian landscapes. Nguyen et al. (31) further showed that interventions targeting temperature-sensitive life stages in schistosomiasis can displace the thermal optimum for transmission by more than 1 degree Celsius, with direct implications for control strategy under warming scenarios.

Sures et al. (4) identified synergistic interactions between habitat alteration, global warming, and chemical pollution as creating multi-stressor scenarios for aquatic parasite communities that are not predictable from single-stressor analyses. Thomas (6) synthesised evidence across infectious disease systems to emphasise the non-linear and bi-directional character of climate-disease relationships: some parasites increase in abundance and range while others decline, depending on their position relative to their thermal optimum and the concurrent responses of their host communities. Carlson et al. (8) projected global-scale parasite extinction and redistribution under high-emissions RCP 8.5 scenarios, estimating that up to ~30% of parasitic worm species face extinction risk by 2070 under host-driven co-extinction scenarios—an upper bound contingent on concurrent host and parasite range losses, compared to a 5–10% baseline from habitat loss alone; this figure is model-dependent and scenario-specific and must not be interpreted as a central or unconditional estimate.

## 4 Parasite dynamics in arid and semi-arid climates

### 4.1 Terrestrial parasite ecology under extreme heat and drought

#### 4.1.1 Evidence geography

Direct Saudi evidence in this section includes the Saudi cryptosporidiosis literature and the Riyadh tick study. Findings from Qatar, Algeria, the wider MENA region, and other settings are used as regional or comparative evidence and are not presented as measurements from Saudi Arabia. Where Saudi-specific data are unavailable, this limitation is stated explicitly.

In arid ecosystems, the interplay between extreme heat, episodic rainfall, and water scarcity creates a distinctive transmission regime for parasites. Drought concentrates both hosts and parasites around diminishing ephemeral hydrological foci, increasing contact rates and faecal-oral transmission of protozoa such as Cryptosporidium and Giardia (32). In water-stressed MENA settings, this concentration pathway compounds epidemiological vulnerability in pastoral and peri-urban populations (32, 33). Areeshi et al. (33) confirmed that cryptosporidiosis is an important cause of diarrhoeal disease in humans and livestock in Saudi Arabia and neighbouring countries, particularly in children and immunocompromised individuals, and identified limited diagnostic infrastructure as a critical constraint on outbreak ascertainment.

Dromedary camels represent a candidate sentinel host in Arabian arid zones (Figure 1D). Sazmand et al. (34) established that only scattered information is available on the full range of zoonotic parasites transmitted to humans via dromedaries, despite known carriage of Cryptosporidium, Giardia, Balantidium, Blastocystis, Enterocytozoon bieneusi, Toxoplasma gondii, Trichinella, *Linguatula serrata*, and Trypanosoma. Abd Alfatah (35) confirmed extreme diversity of gastrointestinal parasitic infestations in dromedaries, with helminth and protozoan infections co-occurring and associated with haematobiochemical disorders consistent with clinically significant parasitism. Maxamhud et al. (36) confirmed through molecular identification that dromedary camels in the Algerian Sahara harbour Cryptosporidium spp. and Giardia duodenalis genotypes with zoonotic transmission potential, establishing verifiable camel-environment-human transmission chains across the desert livestock interface shared with Saudi Arabia.

High-density multi-species agricultural intensification in Saudi Arabia and across the GCC amplifies zoonotic parasite interfaces. Urban ecological conditions in which sanitation infrastructure, food storage practices, and peridomestic habitat structure jointly shape multi-pathogen spillover risk are exemplified by the rodent-borne zoonosis profile documented in Qatar, where peridomestic rodents carry multiple pathogens simultaneously under conditions linked to urbanisation and inadequate farm biosecurity (37). As camel farming intensifies regionally to meet growing demand for camel products, the animal-human interface at farms, markets, and processing facilities expands, increasing the zoonotic exposure surface for the diverse parasite fauna documented by Sazmand et al. (34).

### 4.2 Tick vectors as climate-sensitive bioindicators in hyper-arid systems

Ticks are among the most climate-sensitive arthropod vectors, with activity periods, questing behaviour, and geographic ranges closely coupled to temperature and humidity (21). Sajid et al. (38) reviewed the ecobiology, vector significance, and control of Hyalomma ticks, identifying temperature, climate, host age and sex, and breed as key associated risk factors modulating infestation intensity in camels and livestock across arid zones. In Riyadh Province, Saudi Arabia, Alanazi et al. (39) documented *Hyalomma dromedarii* and Rhipicephalus spp. as the dominant tick species infesting dromedary camels, with collections exhibiting a unimodal thermal activity curve peaking during summer months at 36–39.9% of total tick counts—a phenological signal consistent with the direct thermal modulation of tick questing behaviour described in temperate and Mediterranean systems (21, 42).

Range expansion of Hyalomma species under warming is documented across latitudinal and altitudinal gradients. Estrada-Pena et al. (40) used retrospective climate modelling to show that warming trends have already expanded thermal suitability for *Hyalomma marginatum* into previously marginal areas, and projected further expansion under continued warming. Fernandez-Ruiz and Estrada-Pena (41) confirmed that climate trends increase environmental suitability for permanent *H. marginatum* populations in previously unsuitable European zones—a pattern with direct analogues in the warming highlands of the Arabian Peninsula. Valcárcel et al. (42) demonstrated that both *H. lusitanicum* and *H. marginatum* are currently spreading to new areas due to climate change and host movement, highlighting the synergistic role of anthropogenic host translocation—specifically trans-boundary livestock trade, camel movement, and wildlife migration—as the primary mechanism driving tick dispersal across the MENA landscape. Sesmero-Garcia et al. (43) drew attention to the obligate endosymbiont-tick relationship in Hyalomma: Francisella-like endosymbionts synthesise essential B vitamins required for tick survival, and the thermal tolerance of these endosymbionts may be inextricably linked to the tick’s own thermal expansion potential under warming—whether rising temperatures will uncouple this symbiosis or select for thermotolerant endosymbiont strains with enhanced vectorial capacity in Arabian arid zones remains an unresolved and scientifically important question.

Dust storms, a defining feature of Arabian arid landscapes, may influence parasite transmission ecology through indirect host-mediated pathways, but they should not be presented as an established mechanism for physical dispersal of ixodid ticks. Direct aeolian transport of ixodid ticks is not supported by a demonstrated regional transport mechanism and is aerodynamically implausible relative to the mass-to-drag characteristics of these arthropods. The dominant documented pathway for regional dispersal of Hyalomma and Rhipicephalus is host-mediated, involving transboundary livestock trade, camel movement, and movement of wildlife and other migratory fauna. Dust events may nevertheless alter host physiology, respiratory health, habitat conditions, and coastal nutrient inputs, thereby affecting parasite transmission indirectly. These indirect effects should be distinguished from vector transport itself.

## 5 Aquatic systems: marine parasites in the Red Sea and Arabian Gulf

### 5.1 Fish parasite assemblages as proposed coastal bioindicators

The Red Sea and Arabian Gulf are among the world’s warmest and most saline marine environments, creating naturally extreme conditions for host–parasite interactions and providing spatial analogues for projected future ocean warming. Lewis et al. (19) established that aquatic parasite communities reflect water quality, eutrophication, and contamination history, while Diamant et al. (20) demonstrated that disaggregating fish parasite communities by life-history type—separating macroparasites from microparasites—substantially improved environmental stress detection in the Red Sea context. Palm et al. (18) confirmed parasite diversity as a candidate indicator of long-term environmental change in tropical mariculture systems analogous to Gulf fisheries. Occhibove et al. (44) recently showed that parasite community fine-scale variability in Mediterranean moray eels reflects trophic network stability at spatial scales relevant to local monitoring programmes—a conceptual model transferable to Red Sea reef fish communities. Byers (45) and Atroch et al. (25) emphasised that climate-induced changes in dissolved oxygen, ocean acidity, salinity, and host distributions alter marine parasitism in ways that make fish parasite monitoring a potentially valuable component of integrated ocean health assessment, provided standardised baseline data are established.

In Saudi Red Sea waters, Al-Hasawi (46) demonstrated that intestinal helminth parasites of *Siganus rivulatus* function as accumulation indicators for trace metal pollution, with parasite community structure and metal loads reflecting spatial anthropogenic gradients along the coast. Abdelkhalik et al. (47) confirmed that helminth species richness and diversity indices demonstrate statistically significant correlations with physicochemical water quality parameters in commercial fish from the Jazan coastal area. Hassanine et al. (12) evaluated the acanthocephalan *Sclerocollum saudii* as a sentinel for Red Sea heavy-metal pollution, reporting lead bioconcentration up to 289-fold above host muscle—an extreme accumulation explained mechanistically by the tegumental absorption pathway unique to acanthocephalans, which lack a digestive tract. Baghdadi et al. (13) and Sedaghat et al. (14) demonstrated in *Argyrops spinifer* and *Psettodes erumei, respectively,* that raphidascaridid nematodes also accumulate trace metals above host tissue levels, but via the ingestion route rather than tegumental absorption, yielding different metal profiles and requiring distinct analytical interpretation.

Gefen et al. (15) cautioned that pooled heteroxenous-to-monoxenous indices applied without site-level and host-level stratification are statistically vulnerable to false signal detection. This concern is especially important because parasite abundance data are commonly sparse and zero-inflated; unstratified h/m ratios can therefore be driven by sampling zeros, host composition, site effects, or uneven sampling effort rather than a genuine environmental signal. Accordingly, h/m ratios should be treated as exploratory community indicators unless sampling effort, host characteristics, site, season, and zero-inflated count structure are explicitly incorporated into the analysis. Future surveillance frameworks in the Red Sea and Arabian Gulf should therefore transition from simple univariate h/m ratios to multivariate approaches such as non-metric multidimensional scaling (nMDS) or principal coordinate analysis (PCoA), which can accommodate zero-inflated parasite count data and account for host-specific physiological covariates.

The Arabian Gulf provides direct evidence for temperature-dependent transmission phenology in marine parasites. Ibrahim et al. (48) reported the first record of a novel Kudoa sp. (Myxozoa, Multivalvulida) infecting the purple-spotted bigeye (*Priacanthus tayenus*) at the Jubail landing site in the Saudi Arabian Gulf, with an overall annual infection prevalence of 5.1%, a summer peak of 7.6%, spring prevalence of 6%, autumn prevalence of 2.5%, and zero infections detected in winter. This pronounced temperature-dependent phenological peak in a warm semi-enclosed basin with extreme summer temperatures is consistent with the thermal transmission models of Marcogliese (23, 24) and provides the most temporally resolved evidence currently available for seasonal climate-parasite coupling in Saudi Gulf fisheries. Myxozoan plasmodia in fish skeletal muscle characteristically elicit little inflammatory response, which means that infected fish may reach commercial markets undetected, adding a food safety dimension to the bioindicator monitoring rationale.

### 5.2 Pollution-parasite coupling: mechanistic differentiation and multi-stressor dynamics

The interaction between pollution, environmental stress, and parasitism in coastal waters produces complex signals whose interpretation requires functional distinction between accumulation and effect indicators (2–4). Bayoumy et al. (49) explicitly integrated heavy metal pollution and fish parasite data at the Arabian Gulf off Dammam coast in Saudi Arabia, demonstrating co-occurrence of elevated metal bioaccumulation and elevated parasite burden—a co-stressor pattern consistent with pollution-driven immunosuppression increasing host susceptibility to parasitic infection beyond levels expected from transmission dynamics alone. Alafif et al. (50) determined trace metal concentrations in Siganid fish and their endohelminth parasites at two major Red Sea sites, published in the Journal of King Abdulaziz University – Marine Sciences, confirming that parasites differentially accumulate metals relative to host tissues in patterns that mirror spatial anthropogenic pressure gradients along the Saudi coast.

Sures et al. (2) identified three functional parasite bioindicator categories: accumulation indicators (tegumental absorbers such as acanthocephalans, and ingestion-based accumulators such as nematodes), effect indicators (community composition metrics such as h/m ratios, species richness shifts), and components of multivariate bioindicator suites combining both. The Red Sea and Gulf studies reviewed above provide empirical support for all three categories operating simultaneously in Saudi marine systems. Sures et al. (4) further established that synergistic interactions between warming and chemical pollution create multi-stressor scenarios in which parasite bioindicator signals may require multivariate analytical frameworks to separate the contributions of thermal change, metal loading, and eutrophication to observed community shifts.

## 6 Regional environmental drivers and parasite responses

### 6.1 Desalination, salinity shifts, and waterborne parasites

A testable but currently unvalidated hypothesis is that desalination brine effluents could create local physicochemical conditions that influence the persistence of Cryptosporidium oocysts and Giardia cysts. This proposition is mechanistically plausible only in the limited sense that these environmentally resistant stages can tolerate a range of physicochemical stresses; neither Ben Ayed et al. (51) nor the other cited sources directly demonstrate enhanced persistence or infectivity in desalination-brine mixing zones. Accordingly, the present review does not infer that desalination brine causes parasite persistence or increases public-health risk. Direct evaluation would require controlled salinity-gradient experiments measuring viability/infectivity together with near-shore hydrodynamic measurements or modelling of brine-plume dispersion. Until such data are available, this pathway should be regarded strictly as a hypothesis for future testing.

Across 10 MENA countries, waterborne Cryptosporidium (approximately 24.5% detection rate, 95% CI 16.3–33.8%) and Giardia duodenalis (approximately 37.7%, 95% CI 21.9–55.1%) have been detected in drinking water, recreational water, and wastewater resources, with highest concentrations in raw wastewater and surface waters (51) (Figure 1B). The likely underreporting implied by the absence of formally documented outbreaks despite these contamination levels (51) represents both an epidemiological concern and a monitoring gap that standardised surveillance at desalination plants, wastewater treatment facilities, and irrigation networks would address. Khan et al. (32) reviewed Cryptosporidium and Giardia epidemiology across Middle Eastern countries, identifying water stress, hygiene deficits, and inadequate treatment infrastructure as primary structural drivers of transmission. Areeshi et al. (33) documented cryptosporidiosis as a significant cause of human diarrhoeal disease in Saudi Arabia, particularly in children.

Bourli et al. (52) reviewed 416 protozoan waterborne outbreaks globally between 2017 and 2022, with Cryptosporidium accounting for 77.4% of events and Giardia for 17.1%, predominantly in recreational and swimming-pool water—a finding directly relevant to rapidly expanding Saudi and GCC urban water recreation infrastructure.

### 6.2 Host-mediated vector dispersal, land-use change, and parasite expansion

High-density multi-species agricultural intensification in Saudi Arabia and across the GCC amplifies zoonotic parasite interfaces through the proliferation of mixed species at shared watering and feed points, expansion of peridomestic wildlife habitat, and the concentration of stray animals in peri-urban zones. The geographic spread of Hyalomma and Rhipicephalus ticks in the MENA region is overwhelmingly driven by anthropogenic host translocation—trans-boundary livestock trade, camel movement along established trading and pilgrimage routes, and wildlife migration—rather than passive dispersal mechanisms. Valcárcel et al. (42) demonstrated that both *H. lusitanicum* and *H. marginatum* spread to new areas through the combined action of climate change and host movement, emphasising that surveillance systems for tick range expansion in the Arabian Peninsula must track livestock and camel movement networks alongside meteorological variables. The annual Hajj and Umrah pilgrimages concentrate individuals from diverse endemic regions into confined spaces, creating recurring transboundary pathogen introduction pathways that are unquantified in current surveillance frameworks (7).

### 6.3 Irrigation, artificial water bodies, and helminth transmission foci

Irrigation infrastructure in Saudi Arabia’s agricultural regions creates artificial wetland microhabitats that support intermediate host populations for helminth parasites and breeding sites for vector arthropods. In southwestern Saudi Arabia, residual schistosomiasis transmission persists in focal pockets linked to rainfall, temperature, and snail habitat availability (7). Nguyen et al. (31) modelled schistosomiasis transmission under warming, demonstrating that interventions targeting temperature-sensitive cercarial production can shift the thermal optimum for transmission by more than 1 degree Celsius—implying that control strategies in Saudi wadi systems must account for locally projected temperature increases. The combination of expanding irrigation infrastructure, warming temperatures, and available definitive hosts in Saudi livestock creates conditions broadly analogous to those associated with schistosomiasis persistence in irrigated systems across MENA (7) (Table 1).

**Table 1:** Key parasite–host systems with proposed bioindicator relevance in Saudi Arabia and the Arabian Peninsula, functionally categorised as accumulation indicators or effect indicators.

Parasite/Pathogen	Host	Region	Reported prevalence/Signal	Environmental Driver	References
*Cryptosporidium* spp.; *Giardia duodenalis*	Waterborne (human & livestock)	10 MENA / GCC countries	~24.5% (Cryptosporidium); ~37.7% (Giardia) in water resources	Water scarcity; sanitation deficits; desalination effluents	(32, 51)
*Coxiella burnetii* (bacterial pathogen; not a eukaryotic parasite)	Dromedary camels; livestock; abattoir workers	Saudi Arabia (Makkah); UAE (abattoir)	50.4% individual camel seroprevalence Makkah Province (53); 46.7% individual camel seroprevalence, 92.9% herd-level seropositivity national (54); 52.9% slaughtered camels, 24.4% abattoir workers UAE (56)	Heat stress; drought; high-density processing; tick infestation	(53–56)
*Cryptosporidium* spp., *Giardia* spp.*, Toxoplasma gondii, Linguatula serrata, Trypanosoma* spp. (multi-taxon)	Dromedary camels	Arabian deserts and Sahara	High diversity; widespread zoonotic fauna	Water scarcity; camel farming intensification; close human-animal contact	(34–36)
*Hyalomma dromedarii; Rhipicephalus* spp.	Camels and wild mammals	Riyadh Province, Saudi Arabia	Unimodal thermal activity curve peaking during summer months; 36–39.9% of tick collections	Seasonal heat; host-mediated range expansion	(21, 38, 39)
*Sclerocollum saudii* (Acanthocephala; accumulation indicator: Pb up to 289x host muscle)	Marine fish	Red Sea, Saudi coast	Bioconcentration of lead up to 289-fold above host muscle; accumulation indicator of heavy-metal loading	Industrial trace metal contamination; coastal pollution	(12)
*Hysterothylacium* spp. larvae (nematode; accumulation indicator via intestinal ingestion route)	*Psettodes erumei*; *Argyrops spinifer*	Persian Gulf; Red Sea	Differential trace metal accumulation via ingestion above host tissues	Industrial metal loading; coastal pollution	(13, 14)
Kudoa sp. (Myxozoa, Multivalvulida; effect indicator via thermal transmission phenology)	*Priacanthus tayenus* (purple-spotted bigeye)	Jubail landing site, Saudi Arabian Gulf	Annual prevalence 5.1%; summer peak 7.6%; spring 6%; autumn 2.5%; absent in winter — temperature-dependent phenological peak	Seasonal thermal shifts; warm semi-enclosed Gulf waters	(48)
Heteroxenous-to-monoxenous parasite ratio (community index; effect indicator)	*Siganus rivulatus*; coral reef fish	Gulf of Aqaba, Red Sea	Directional trophic shift: suppression of heteroxenous taxa concurrent with proliferation of monoxenous taxa at impacted sites	Anthropogenic habitat degradation; loss of benthic intermediate hosts	(9, 10, 15)
Human taeniosis (predominantly T. saginata; confirmed in 11/21 MENA countries)	Humans; bovine intermediate host	MENA region (18/21 countries)	86% (18/21) human taeniosis; T. saginata explicitly identified in 11/21; bovine cysticercosis data critically sparse	Livestock management; climate stress on transmission	(57)

## 7 Zoonotic parasites and one health in the Arabian peninsula

### 7.1 Camel-human zoonotic networks and *Coxiella burnetii*: epidemiological stratification and taxonomic scope

The One Health paradigm recognises the structural interdependence of human, animal, and environmental health—a framework particularly apt for understanding parasite bioindicator signals in arid regions, where the same environmental stressors that alter parasite communities can simultaneously influence zoonotic pathogen transmission. *Coxiella burnetii* is included here as a zoonotic bacterial pathogen, not as a eukaryotic parasite. Taxonomically, *C. burnetii* is an obligate intracellular Gram-negative bacterium and the causative agent of Q fever. Its inclusion is intended to broaden the One Health context of the review and to illustrate how environmental, livestock, occupational, and pathogen-surveillance signals can intersect; it is not classified as a parasite or used as evidence for parasite-specific bioindicator function. Dromedary camels serve as reservoirs for MERS-CoV, Q fever, and a diverse zoonotic parasite fauna whose transmission is modulated by environmental conditions, animal management practices, and human–animal contact patterns. Epidemiological interpretation of *C. burnetii* seroprevalence in the region requires careful stratification across distinct measurement levels that are frequently conflated in the literature.

At the individual animal level, Alkenani et al. (53) conducted a serological investigation of *C. burnetii* infection in livestock across Makkah Province, Saudi Arabia (n = 788 sera; indirect ELISA for anti-*C. burnetii* IgG antibodies), documenting a camel seroprevalence of 50.4%—the highest across all tested livestock species in that study, reflecting serological exposure (past or current) rather than active infection, which the same group quantified at substantially lower levels (2.5% blood; 6.6% vaginal swabs) by real-time PCR. At the herd level, Kasem et al. (54) reported nationwide *C. burnetii* seroprevalence across Saudi farm animals across 7,760 sera, documenting an individual-animal camel seroprevalence of 46.7% and a camel herd-level seropositivity reaching 92.9%—a figure that integrates individual variation and indicates near-universal herd exposure nationally. These three metrics must not be conflated: Alkenani et al. (53) measured individual seroprevalence (~50.4%) in Makkah Province specifically; Kasem et al. (54) independently reported both individual (~46.7%) and herd-level (~92.9%) seropositivity nationwide. Each represents a fundamentally different epidemiological denominator and geographic scope and must not be combined or substituted for one another. Devaux et al. (55) synthesised evidence that *C. burnetii* persists in camel adipose tissue, implying that, once infected, herds may remain reservoirs for extended periods, with environmental heat and drought stress potentially exacerbating bacterial shedding through reproductive failure.

At the occupational exposure level, Sheek-Hussein et al. (56) provided direct One Health evidence from UAE abattoir settings: 52.9% seroprevalence in slaughtered camels and 24.4% seropositivity in abattoir workers processing those animals, despite negative blood qPCR in all tested workers, demonstrating substantive occupational exposure to *C. burnetii* in high-density livestock processing environments characteristic of GCC urban-industrial settings. The causal pathway from high individual-animal seroprevalence through occupational aerosol exposure to human seroconversion is thereby empirically documented in a Gulf arid-zone context.

### 7.2 Neglected tropical diseases: epidemiological context and climate threats

The MENA region carries a substantial burden of neglected tropical diseases (NTDs) including schistosomiasis, leishmaniasis, echinococcosis, fascioliasis, and brucellosis, with disproportionate impact on low-income populations (7). While several GCC states have achieved NTD control milestones through mass drug administration and vector control programmes (5), these gains face directionally context-dependent threats from climate change that may, in some systems, expand vector ranges while in others disrupting established transmission cycles. Warming temperatures may expand the geographic suitability of sandfly vectors for Leishmania species in previously unsuitable highland zones—a risk consistent with leishmaniasis range dynamics documented in neighbouring MENA countries (7). Human taeniosis—predominantly attributable to Taenia saginata given regional pork-prohibition patterns—has been confirmed in 86% (18/21) of MENA countries, with T. saginata explicitly identified in 11/21; bovine cysticercosis data remain critically sparse, representing a surveillance gap for a parasite whose transmission is modulated by livestock management practices increasingly stressed by climate and economic pressures (57).

### 7.3 Waterborne protozoa at the livestock-human Interface

#### 7.3.1 Evidence geography

Areeshi et al. (33) provides direct Saudi evidence concerning human cryptosporidiosis. The MENA-wide synthesis of Ben Ayed et al. (51), the global outbreak analysis of Bourli et al. (52), and molecular studies from other MENA countries provide regional or international context. These studies support plausibility and comparative interpretation but do not establish site-specific transmission or desalination-associated risk in Saudi Arabia.

Cryptosporidiosis is a growing concern in GCC states, with the burden falling disproportionately on children and immunocompromised individuals (33). Bourli et al. (52) documented the global outbreak profile and identified the absence of formally reported outbreaks in MENA despite high documented contamination as likely reflecting systematic underreporting rather than absence of disease. Integrating Cryptosporidium oocyst and Giardia cyst quantification—with molecular genotyping for source attribution—into routine water-quality testing at desalination plants, wastewater treatment facilities, and irrigation networks would yield dual benefits: protecting public health and generating continuous, structured bioindicator data on waterborne parasite pressure in arid water systems. Molecular genotyping studies across MENA irrigated agricultural systems consistently identify shared genotypes between livestock and irrigation water, establishing the plausibility of verifiable animal-to-environment-to-human transmission chains applicable to Saudi irrigated zones; site-specific data from Saudi Arabia itself remain a priority research gap.

## 8 Marine parasite bioindicators in Saudi waters: integrative assessment

### 8.1 Evidence geography

The marine evidence is separated into direct Saudi observations, regional evidence from the Gulf of Aqaba/Jordan, and extra-regional comparative studies. Comparative evidence is used to support mechanisms or methodological approaches rather than to substitute for Saudi longitudinal validation.

The Red Sea and Arabian Gulf provide contrasting marine environments for evaluating parasite bioindicator systems. The Red Sea, with its pronounced latitudinal thermal and salinity gradients, high endemism, and extensive coral reef systems, supports parasite communities whose composition reflects fine-scale habitat heterogeneity. Dzikowski et al. (10) documented decade-scale declines in the heteroxenous-to-monoxenous parasite ratio in rabbitfish communities in the Gulf of Aqaba, interpreted as reflecting progressive food-web simplification driven by benthic intermediate host loss under anthropogenic pressure. Al-Zibdah et al. (9) corroborated this trophic shift pattern in coral reef fish at impacted coastal sites. The integrated evidence from Al-Hasawi (46), Hassanine et al. (12), Baghdadi et al. (13), Alafif et al. (50), and Abdelkhalik et al. (47) collectively demonstrates that Saudi Red Sea fish parasites function simultaneously as accumulation indicators and as effect indicators of anthropogenic coastal stress—two functionally distinct properties that require different monitoring protocols and analytical frameworks to exploit.

The Arabian Gulf, a shallow semi-enclosed basin with summer surface temperatures regularly exceeding 35 degrees Celsius, functions as a natural analogue for projected future ocean warming in other tropical systems. Ibrahim et al. (48) provided the first and currently only temporally resolved evidence for temperature-dependent transmission phenology in Saudi Gulf fish parasites—the summer prevalence peak of Kudoa sp. in *Priacanthus tayenus* at Jubail—demonstrating that myxozoan infection follows a seasonal pattern consistent with thermal modulation. Bayoumy et al. (49) integrated parasitological and toxicological measurements in the Gulf off Dammam, affirming the value of multi-stressor assessments in which parasite burden and trace metal load are measured simultaneously. A One Health approach to marine bioindicator monitoring should integrate parasitological, toxicological, and microbiological sampling within unified fishery surveillance programmes, providing actionable intelligence on both ecosystem health and food safety, particularly given that myxozoan-infected fish may reach commercial markets without detection.

Despite this evidence base, the proposed bioindicator value of these systems remains incompletely validated. Current data are predominantly cross-sectional and confined to a small number of commercially important fish species, constraining ecological interpretation and precluding detection of directional change. The Red Sea’s latitudinal thermal gradient offers a spatial-for-temporal substitution design that could accelerate bioindicator calibration even before long time series become available (22). Occhibove et al. (44) demonstrated in Mediterranean systems that community-level parasite variability captures trophic network stability at local scales, suggesting that similar multi-taxon assemblage approaches in Red Sea reef fish could provide integrated ecosystem health metrics beyond the capability of any single bioindicator taxon.

## 9 Research gaps and priorities

Operationalising parasites as environmental bioindicators in Saudi Arabia is constrained by a set of interrelated data and methodological gaps summarised in Table 2. The most fundamental limitation is the absence of long-term temporal datasets linking parasite community data to climatic and environmental variables at fixed sentinel sites. Without multi-year time series, directional trends cannot be separated from stochastic inter-annual variation—a limitation affecting terrestrial tick assemblages (39), camel-associated zoonoses (55), and marine fish parasite communities (9, 46, 48) alike. A second gap is the absence of mechanistic evidence for the desalination-waterborne protozoa hypothesis: whether elevated brine salinity at mixing zones actively preserves Cryptosporidium and Giardia infectivity or merely delays degradation requires controlled halotolerance experiments combined with near-shore hydrodynamic modelling of brine plume dispersal and concentration dynamics. Third, the shift from univariate h/m ratios to multivariate community indices (nMDS, PCoA) in Red Sea and Gulf monitoring programmes would substantially improve the statistical robustness of bioindicator assessments by accommodating zero-inflated count data and host-specific covariates. Fourth, the host-mediated dispersal pathways for Hyalomma and Rhipicephalus ticks via trans-boundary livestock and camel trade are under-quantified, and molecular epidemiology of tick strains across Saudi livestock movement networks is largely absent.

**Table 2:** Critical research gaps and priority methodological approaches for parasite bioindicator development in Saudi Arabia.

Research gap	Consequence	Priority approach
Absence of long-term parasite time series in Saudi Arabia	Cannot distinguish directional trends from stochastic variation	Sentinel monitoring with standardised annual sampling at fixed sites
No coupled climate-parasite–host datasets	Restricts causal inference; limits mechanistic modelling	Paired meteorological, host demographic, and parasitological data collection
Sparse Red Sea and Gulf marine parasite baselines	Before-after comparisons of climate-driven compositional change impossible	Molecular baseline surveys at sentinel reef and fishery sites; shift from univariate h/m ratios to multivariate nMDS/PCoA frameworks
Scarce data on host-mediated vector dispersal (Hyalomma, Rhipicephalus) via livestock and camel trade routes	Under-quantified pathway of tick range expansion onto Arabian highlands	Integration of livestock movement tracking, tick phenology, and molecular epidemiology
Limited molecular genotyping of waterborne protozoa	Insufficient source attribution for Cryptosporidium and Giardia	NGS of environmental and clinical samples across GCC states
Sparse bovine cysticercosis and Taenia saginata transmission data	Human taeniosis confirmed in 86% (18/21) MENA countries; T. saginata explicitly identified in 11/21; transmission burden and bovine cysticercosis data critically sparse	Abattoir-based surveillance linked to livestock origin and climate data
Absent data on Cryptosporidium oocyst halotolerance thresholds and osmotic resilience in brine effluent mixing zones	Desalination-waterborne protozoa hypothesis remains untested	Controlled halotolerance experiments combined with near-shore hydrodynamic modelling of brine plume dispersal

## 10 Towards a surveillance framework for parasite-based environmental monitoring

Building on the evidence reviewed, an operational surveillance framework for parasite-based environmental monitoring in Saudi Arabia requires five complementary components. First, terrestrial sentinel monitoring: annual parasitological surveys of dromedary camels at major livestock markets and herding corridors, peridomestic rodents in expanding urban fringes, and tick vectors at urban-wildland interfaces, using standardised collection protocols linked to co-located meteorological records, with tick collections stratified by season to characterise unimodal thermal activity curves (34, 37, 39). Second, aquatic and marine bioindication: baseline and repeat parasite community surveys at sentinel Red Sea reef sites and Arabian Gulf fishery landing sites (including Jubail), incorporating both morphological taxonomy and molecular identification, using multivariate community indices (nMDS, PCoA) rather than simple h/m ratios, and embedded within existing marine biodiversity and water-quality monitoring programmes (4, 46, 48). Third, waterborne parasite integration: addition of Cryptosporidium oocyst and Giardia cyst quantification—with species-level molecular genotyping for source attribution—to routine water-quality testing at desalination plants, wastewater treatment facilities, and irrigation networks (51, 52). Fourth, One Health data integration: shared veterinary-medical-environmental databases linking parasite, host, and climatic data within a geospatial framework, stratified by individual-level and herd-level metrics, enabling cross-sector zoonotic risk analysis (54–56). Fifth, technology integration: remote sensing for land-use change and vegetation monitoring, livestock movement tracking for host-mediated tick dispersal mapping, and molecular epidemiological strain typing to develop predictive models of transmission risk under plausible climate scenarios (6, 8).

## 11 Discussion

The evidence reviewed across terrestrial, aquatic, and zoonotic domains supports the view that parasites in Saudi Arabia and the Arabian Peninsula are responding to environmental change in ways that are detectable with current methods—and that systematic monitoring of these responses constitutes a scientifically defensible, though not yet longitudinally validated, early-warning surveillance strategy. Several lines of converging evidence underpin this assessment: dromedaries with high individual-level *C. burnetii* seroprevalence [50.4% in Makkah Province; (53); 46.7% individual seroprevalence nationally; (54)] and near-universal herd-level exposure nationally (92.9%) (54), diverse zoonotic parasite fauna (34), ticks exhibiting unimodal thermal activity curves peaking in summer (39), waterborne protozoa in approximately 24.5–37.7% of MENA water resources (51), and marine fish parasites demonstrating trace metal bioaccumulation and temperature-dependent transmission seasonality (12, 48). Taken together, these constitute a multi-domain bioindicator signal, not a collection of isolated observations.

Mechanistic interpretation must, however, be calibrated against the limitations of the available evidence. The most robustly supported pathways in the current literature are thermal modulation of parasite and vector phenology—consistent with Estrada-Pena et al. (21), Marcogliese (24), and the observed summer activity peaks in both ticks (39) and Kudoa sp. (48)—and host–parasite concentration around ephemeral hydrological foci during drought (32, 51). The salinity-selection pathway for Cryptosporidium and Giardia around desalination outfalls, while mechanistically plausible given the osmotic resilience of cyst walls, has not been empirically demonstrated in Saudi coastal settings and should be classified as a testable hypothesis pending controlled halotolerance experiments. Attribution of observed parasite patterns specifically to climate change, as distinct from concurrent anthropogenic drivers including high-density agricultural intensification, urbanisation, and improved diagnostic ascertainment, remains difficult without long-term coupled datasets—a limitation shared across the regional literature (11, 23).

The thermal ecology framework demands explicit acknowledgement of non-linearity and bidirectionality. Critically, warming does not uniformly amplify parasitism: Gehman et al. (29) demonstrated that warming of as little as ~2 °C can drive local extirpation of thermally sensitive parasite taxa in some coastal systems while amplifying transmission in others—a result that is fully symmetric with, and equally weighted against, any pro-parasitic amplification narrative. Tomamichel et al. (26) established through meta-analysis that temperature-amplified parasite-induced host mortality varies substantially by host order and parasite type, and notably that bacterial parasites showed reduced infected-host mortality with warming—an anti-amplification signal that must receive symmetric emphasis alongside the overall warming–mortality correlation. Studer et al. (27) experimentally demonstrated a unimodal transmission curve with reduced cercarial output and infectivity at temperatures exceeding 30 °C. These findings collectively preclude deterministic projections that warming will uniformly increase parasitism, and require that climate-parasite predictions be stated as directionally context-dependent and highly variable across systems. The 5-degree Celsius shift in the thermal optimum for mosquito-borne parasite transmission demonstrated by Dahlin et al. (30) under ecologically simplified host conditions—conditions characteristic of degraded arid-zone habitats—represents the most quantitatively precise mechanism by which host biodiversity loss could reconFigure transmission dynamics at temperatures not predicted by single-species thermal models.

The marine synthesis was also critically screened to ensure that primary studies were matched to the claims they directly support. In particular, the seasonal Kudoa sp. evidence is attributed to Ibrahim et al. (48), whereas Sedaghat et al. (14) is cited specifically for trace-metal accumulation in Hysterothylacium spp. Barakat et al. (58) is used only as contextual evidence of anthropogenic stress in Gulf of Aqaba reef habitats.

Overall, the review supports a cautious interpretation: parasite-based environmental monitoring has a credible scientific basis, particularly when parasite accumulation and community-level effect indicators are analysed alongside environmental covariates. However, the transition from promising ecological signal to validated early warning requires prospective, standardised and longitudinal evaluation. The specific implementation priorities are detailed separately in Section 9 and the surveillance architecture is presented in Section 10.

## 12 Conclusion

Parasites and parasite communities are promising candidate indicators of environmental change in arid and coastal ecosystems, but the evidence reviewed here does not yet justify treating them as validated early-warning systems. Saudi Arabian studies provide direct examples of trace-metal accumulation by fish parasites, seasonal parasite phenology, tick activity, and zoonotic exposure, while regional and extra-regional studies provide mechanistic context. The principal limitation is the scarcity of long-term, standardised, coupled datasets capable of separating climate effects from pollution, land-use change, host movement, and other concurrent drivers. Future progress therefore depends on longitudinal sentinel surveillance that combines parasitological measurements with environmental, climatic, host, movement, and molecular data. The proposed framework should be considered a research and surveillance architecture requiring field validation rather than an already operational monitoring system.
